# COVID-19 pandemic preparation: using simulation for systems-based learning to prepare the largest healthcare workforce and system in Canada

**DOI:** 10.1186/s41077-020-00138-w

**Published:** 2020-08-18

**Authors:** Mirette Dubé, Alyshah Kaba, Theresa Cronin, Sue Barnes, Tara Fuselli, Vincent Grant

**Affiliations:** 1 eSIM Provincial Simulation Program, Alberta Health Services, Alberta Health Services, 1403 29th Street NW, Calgary, Alberta T2N 2 T9 Canada; 2 Department of Community Health Sciences, Cumming School of Medicine, University of Calgary, Calgary, Canada; 3 Departments of Pediatrics and Emergency Medicine, Cumming School of Medicine, University of Calgary, Calgary, Canada; 4 KidSIM Pediatric Simulation Program, Alberta Children’s Hospital, 28 Oki Dr NW, Calgary, Alberta T3B 6A8 Canada

**Keywords:** Simulation, COVID-19; debriefing, Systems integration, Pandemic preparation, Quality, Safety, Organizational learning

## Abstract

Healthcare resources have been strained to previously unforeseeable limits as a result of the COVID-19 pandemic of 2020. This has prompted the emergence of critical just-in-time COVID-19 education, including rapid simulation preparedness, evaluation and training across all healthcare sectors. Simulation has been proven to be pivotal for both healthcare provider learning and systems integration in the context of testing and integrating new processes, workflows, and rapid changes to practice (e.g., new cognitive aids, checklists, protocols) and changes to the delivery of clinical care. The individual, team, and systems learnings generated from proactive simulation training is occurring at unprecedented volume and speed in our healthcare system. Establishing a clear process to collect and report simulation outcomes has never been more important for staff and patient safety to reduce preventable harm. Our provincial simulation program in the province of Alberta, Canada (population = 4.37 million; geographic area = 661,848 km^2^), has rapidly responded to this need by leading the intake, design, development, planning, and co-facilitation of over 400 acute care simulations across our province in both urban and rural Emergency Departments, Intensive Care Units, Operating Rooms, Labor and Delivery Units, Urgent Care Centers, Diagnostic Imaging and In-patient Units over a 5-week period to an estimated 30,000 learners of real frontline team members. Unfortunately, the speed at which the COVID-19 pandemic has emerged in Canada may prevent healthcare sectors in both urban and rural settings to have an opportunity for healthcare teams to participate in just-in-time in situ simulation-based learning prior to a potential surge of COVID-19 patients. Our coordinated approach and infrastructure have enabled organizational learnings and the ability to theme and categorize a mass volume of simulation outcome data, primarily from acute care settings to help all sectors further anticipate and plan. The goal of this paper is to share the unique features and advantages of using a centralized provincial simulation response team, preparedness using learning and systems integration methods, and to share the highest risk and highest frequency outcomes from analyzing a mass volume of COVID-19 simulation data across the largest health authority in Canada.

## Introduction

With the emergence of the COVID-19 pandemic of 2020, healthcare resources have been strained to unforeseeable capacities, promoting the need for rapid, effective, and efficient preparedness. This has prompted the emergence of just-in-time preparedness strategies, including simulation for systems evaluation and healthcare provider (HCP) learning to support planning across healthcare sectors [1–9]. Simulation has been pivotal for systems testing and integrating new and improved components such as novel workflows, protocols, and cognitive aids with rapid changes to practice and care delivery. Finding clear approaches to rapidly collect and report what is working well and what needs to change is urgently required for HCP and patient safety to ultimately reduce preventable harm.

The provincial simulation program, named eSIM (educate, simulate, innovate, motivate) in the province of Alberta, Canada (population = 4.37 million; geographic area = 661,848 km^2^, [10]), has rapidly responded to preparedness training by establishing a central eSIM provincial COVID response team (similar to an emergency command center operations team for simulation) to facilitate a large-scale project managing the initiation, planning, execution, and performance/monitoring of a provincial COVID-19 simulation response [11]. Over just 5 weeks, this response team enabled a harmonized intake process, design, and development of a robust COVID-19 simulation curriculum, mobilized a data collection/outcome reporting team, and a response plan to facilitate over 400 acute care simulation session requests across Alberta’s broad geographical zones. This coordinated approach and infrastructure enabled an integrated provincial multi-site simulation response, allowing the ability to rapidly theme and categorize a mass number of simulation findings (over 2,500 systems issues) from over 30,000 learners (HCP) on the frontline. The simulation “command center” served to disseminate the outcomes across a large single health authority to further support on-going planning, develop and refine the curriculum, and remain a specialized contact team of simulation experts for all health sectors (primarily acute care; urban and rural).

The goal of this paper is to share the unique features and advantages of using a centralized provincial simulation response team, preparedness using learning and systems integration methods, and to share the highest risk and highest frequency outcomes from analyzing a mass volume of COVID-19 simulation data across the largest health authority in Canada.

## Background

### Alberta Health Services: largest integrated healthcare system in Canada

Alberta Health Services (AHS) has a centralized approach to its leadership and core operational functions (e.g., human resources and information technology) and provides medical care at over 650 facilities across the province, including 106 acute care hospitals and 25,653 continuing care beds/spaces, five stand-alone psychiatric facilities, 2723 addiction and mental health beds and 243 community palliative and hospice beds. AHS has over 125,000 employees and over 10,000 physicians and service accountability zones are divided into five geographical areas: North, Edmonton, Central, Calgary, and South [10]. There are few organizations in the world, which are comparable to AHS’ integrated healthcare system relative to its size and capacity [12], making it a unique opportunity to glean insights and lessons learned from COVID-19 pandemic preparedness.

### Pandemic preparedness and planning: role of simulation for team training and systems integration

While disaster planning has become core content within some training curricula, the majority of health professionals remain ill prepared to deal with large scale disasters and will not encounter these critical scenarios in practice [13, 14]. Simulation is one method that has been identified to improve readiness and preparedness in times of disaster through deliberate practice and exposure to rare situations [15–18]. Simulation-based learning has historically focused on individual and team training of practicing HCPs [19–25], although it has evolved to include just-in-time in situ training within the actual clinical environment [26–28] including simulation for systems integration (SIS), targeting the testing and integration of systems and processes (e.g., workflows, care pathways) that are uniquely important to disaster preparedness [29–32].

The eSIM COVID-19 response team has been involved in pioneering work in SIS [11, 32, 33] systems integration, an engineering term defined as bringing many subsystems together into one better functioning system, has the potential to improve the safety and quality of care through re-engineering of the processes and systems in which HCP work [34]. The system engineering initiative for patient safety (SEIPS) 2.0 model provides a framework outlining how the work system components (e.g., tools/technology, tasks, environment, people/teams) provide a matrix for thematically categorizing system-focused debriefing (SFD) outcomes in a complex adaptive healthcare system [35]. SIS/SFD allows testing of new processes within healthcare systems to inform design and utility, and to identify system issues proactively to prevent harm [15, 19–21].

The rapid onset of the COVID-19 crisis is having a profound impact on global health, increasing demand, and risks on healthcare systems due to rapidly changing and unpredictable circumstances. The use of in situ simulation as a proactive risk mitigation strategy to prepare healthcare organizations for pandemic planning is well supported in the literature [8, 9, 26–28]. Pandemics place high demands on clinical care and potential risk of contamination for HCP, which increases the fear of spread to others [1, 36, 37]. Deliberate practice through simulation can reduce the cognitive load of HCP involved in direct frontline patient care, potentially mitigating latent safety threats (LSTs) (e.g., errors in design, organization, and/or training that may have a significant impact on patient safety) in times of extended pressure, exhaustion, and burnout [38].

As recently illustrated in both Italy [2] and Singapore [3], simulation is key to preparedness by optimizing work flow structures, developing new processes, managing staffing levels, procuring equipment, bed management, and enforcing consistency of medical management of patients. In those ways, simulation can be used as both a learning and evaluation tool (e.g., SIS/SFD) [32]. Diekmann et al. also described the potential of using simulation to improve hospital responses to the COVID-19 crisis [6]. Recently published COVID-19 simulation papers [8, 9] share lessons learned in an attempt to support organizations that may seek to use simulation for diagnosing, testing, and embedding these approaches within pandemic constraints. While these papers do not provide specific outcomes from their system simulations, they share practical tips, tools, scenarios, debriefing questions, and resources which can be used to analyze the current needs and responses to potentially mitigate the negative impacts of the COVID-19 crisis. Similarly, Chan and Nickson [7], published an example of practical considerations for organizations in the development for just-in-time simulation training, including the scenario development, prebriefing, and debriefing, using system and process simulation for the testing of airway management for suspected/confirmed cases of COVID-19. While many of the findings in the emergent literature above glean insights into the use of simulation as both a learning and evaluation tool to prepare for COVID-19, a key gap identified is that many of these outcomes are unique to the experience of one specific site or one unique institution, which limit the generalizability and scalability of the findings. This is especially important when using simulation for pandemic preparedness to rapidly test systems and processes and prepare frontline teams to care for potential COVID-19 patients.

Therefore, an identified need in the literature is the ability to proactively identify systems issues, while testing new pandemic processes in real time, and sharing these organizational learnings and system-level outcomes on a mass scale to both anticipate and plan for COVID-19. This is an invaluable opportunity to support healthcare organizations’ preparedness during a global pandemic.

## COVID-19 simulation project approach

Our provincial simulation program rapidly mobilized to respond to our organization’s need by establishing a central simulation COVID response team and a large-scale evaluation project to manage the initiation, planning, execution, and performance/monitoring for all simulation-based requests. The following sections will summarize our project evaluation approach based on common project phases [39]: (A) project initiation and planning; (B) project execution; (C) project performance and monitoring; (D) organizational learning; and (E) reporting of outcomes.

## Project initiation and planning

Rapidly designating the eSIM COVID-19 response team allowed a centralized contact and triage system across the province for all simulation-based requests coming from hundreds of centers, allowing coordination, planning, and resource oversight for simulation training. Team members included a team lead, geographically dispersed eSIM consultants (clinicians whose workplace role is to lead the design, delivery, faculty development, and evaluation of simulation methodologies and projects at AHS) and a designated data and outcomes team solely responsible for theming and categorizing thousands of simulation data points from COVID-19 simulations.

The simulation infrastructure in place in AHS prior to COVID-19 (e.g., eSIM consultants positioned in every zone (rural, urban, and mobile program) and > 1300 eSIM-trained simulation educators across AHS) ensured every geographical zone was supported. The intake process was maintained through a central spreadsheet accessible to the entire provincial team and a needs assessment facilitated by an eSIM consultant (Additional file 1 intake form) to ensure the needs and objectives aligned with established simulation-based methodologies and capacity. Requests were prioritized as they were processed and whenever possible, prior relationships with simulation-trained educators comfortable with the use of simulation-based methods were leveraged to help facilitate the large number of sessions. eSIM consultants either fully led the sessions, initiated sessions while mentoring others to replicate delivery on an on-going basis (especially when large numbers of HCP were involved), and/or shared resources with faculty who had the capacity and experience to lead sessions independently.

The first wave of intake requests (and patients) in Alberta, organically flooded from Emergency Departments, followed by Intensive Care Units, Operating Rooms, Labor and Delivery Units, and Neonatal ICUs in Calgary. Simulations were triaged for COVID-19 training and preparedness for the highest risk locations and activities across acute care. A second wave of requests included diagnostic imaging and inpatient medical units (including newly created inpatient units for COVID-19, among others). A similar pattern of requests followed from all of the five geographic zones in Alberta in concordance with the number of COVID-19 cases starting to move through the system. Daily response team meetings ensured strategic planning with eSIM consultants to then specifically target and reach out to acute care sites with no simulation trained champion, those who had not made any requests, and to ensure simulation support and curriculum resources were shared across all zones.

### Curriculum development

A novel part of our approach was the creation of a robust, centrally located simulation curriculum, which was rapidly developed and mobilized over a 10-day period at the onset of the COVID-19 simulation response. Curriculum content was vetted through medical experts, clinical leaders, Infection Prevention and Control consultants, and the organization’s Emergency Command Center to ensure accuracy, alignment, and validity before application. New emerging curriculum resources for COVID-19 were reviewed daily to ensure the most relevant up-to-date information was reflected within current best practice recommendations. The curriculum included COVID-19 simulation scenarios for all sectors, and included a prebriefing script, debriefing tools [32], cognitive aids, “how-to” guides and shared webinars. Curricular materials were shared over the 5-week period with over 600 teams across AHS.

Objectives for the COVID-19 simulation scenarios were based on the highest risk and highest impact system issues identified and prioritized by integrated interprofessional teams of clinicians, managers, and educators—based on their units/departments greatest needs. Table 1 provides samples of frequently used scenario objectives across all simulations and their related work system categories [35].

**Table 1:** Sample COVID-19 simulation scenario objectives (highest risk and highest frequency)

Examples of some COVID-19 scenario objectives (pre-determined to be highest risk and highest frequency)	System issue categories [35]
Equipment: Implement creation of carts for personal protective equipment/intubation Cognitive aids: Apply airway pause with COVID-19 additions Paging systems: Assess addition of “COVID-19” stem to pages to ensure HCP safety and priority response	**Tools and technology**
Task complexity: Prepare smaller specialized teams for intubation (e.g., airway response team) Prepare smaller teams to anticipate and prepare for any aerosol-generating medical procedure Designate and prepare personal protective equipment (PPE) coach for donning and doffing Organize clean runner role to help with cognitive overload	**People and tasks**
Signage: Evaluate plan for new areas for COVID-19 vs non-COVID-19 patients (surge specific phases) Transport routes: Assess, plan, testing of dedicated hallways and elevators	**Environment**
Assess staffing during day and night with Intensive Care Unit surge bed plans and escalating to 2 patients per room Site capacity and triggers: Evaluate pandemic surge exercises identifying triggers, bed capacity, flow restrictions, and continuity across the site	**Organization**
Communication pathways: Apply testing of handover tools and new alerts Workflow: Identify appropriate number staff/roles caring for patient(s) Policies (testing of new or existing): Appraise any unnecessary and preventable delays in care and/or process	**Processes**

## Project execution

Central to our unique approach was the planning and execution of three common simulation-based methods to prepare teams and the healthcare system during COVID-19 preparedness (Table 2). These methods include (1) surge planning and table top debriefing; (2) process walkthrough and environmental scans; and (3) rapid cycle simulation and debriefing. Both learner-focused and system-focused debriefing approaches were used across these three methods [22, 32].

**Table 2:** Method, objective, and examples of intake requests

Method	Objective(s)	Sample of intake requests
Table top exercises	• Surge planning • Bed capacity allocation	• Assess a hospital’s emergency response operational plan related to current bed capacity and pandemic surge planning phases 1 through 4. • Align organizational pandemic policies with current local current state resources to bridge expectations of front line staff and pandemic policy makers.
Process walk-through/environmental scan with debriefing	• Identify LSTs, new workflows, new processes, using a systems approach to debriefing	• Determine new processes for a COVID stroke patient from CT (computed tomography) scan to emergency intubation to angiography. • Determine workflow process from the emergency room triage through to an isolation room for a team COVID-19 intubation process. • Determine the environmental layout and associated workflows, including LSTs when ventilating two patients in one Intensive Care Unit room (new processes, staffing ratios), testing of IT-related issues and central alarm bank. • Determining new and unique processes and educational needs within indigenous health centers and communities to ensure safety of vulnerable populations and ensuring preparedness.
Rapid cycle simulation and debriefing	• Training small groups of interprofessional teams in rapid cycle simulation training and debriefing (once processes are established). Most often 20-min simulations followed by 20-min debriefings.	• Apply new processes of patient flow from triage to isolation room including intubation, safe PPE processes for all emergency departments in Calgary over 2 days (4 adult sites).^a^ • Apply COVID-19 medical management to a complex medical scenario.

^a^Approximately 250 interprofessional team members per site (including physicians, nurses, respiratory therapists, infection prevention and control (IPAC) staff, environmental services, clinical leaders, and other non-clinical team members such as protective services)

### Surge planning and table top debriefing

In some instances, eSIM consultants and human factors (HF) specialists led the co-facilitation and debriefing of table top surge planning exercises. These exercises allowed for proactive pandemic surge planning assessment of facility, departments, programs, and services related to COVID-19 patient flow. eSIM and HF were then utilized for designing decision algorithms, observation of new work processes and work environments, and identifying further improvement opportunities.

### Process walkthrough and environmental scans

Early preparatory work focused on using a systems approach to co-design new COVID-19 processes and spaces. These physical walkthrough simulations were based on day-to-day movement, case-specific patient presentations, and workflows that would be impacted by COVID-19. They were also used to train multiple HCPs to orientate them to the new COVID-19 response for their areas (see Additional file 2 for key points to consider in a process walkthrough environmental scan planning exercise). The primary focus was on identifying LSTs prior to the first patient experience which allowed for application of a new processes in a plan, do, study, act (PDSA) cycle [40]. The team moved through a department/clinical area, doing an environmental scan for barriers, identification of missing equipment, testing of communication pathways, and identification of new tasks/roles/responsibilities. Any changes to the space and processes were actioned to leadership.

### Rapid cycle simulation and debriefing

Once final COVID-19 processes and workflows were established, departments/clinical areas progressed to rapid cycle simulation training followed by debriefing [41]. This involved rapid training sessions (average 20-min scenario duration followed by 20-min debriefings) to ensure small groups of interprofessional team members were able to apply new processes in the live clinical environment prior to use with actual patients. All questions related to medical management, infection, prevention and control (IPAC) and process were also debriefed during these sessions.

## Project performance and monitoring

### Data collection and analysis

**Fig. 1:**
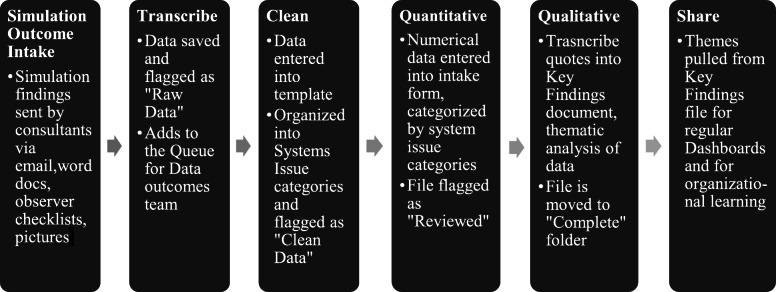
Systematic process for intake and entry of data

A data outcomes team was designated to rapidly process and analyze data collected from all provincial COVID-19 simulations. Figure 1 outlines a systematic iterative process developed for intake and entry of data. An initial coding scheme for categorization of data was created. Data analysis included the theming of system-focused debriefing outcomes [32] based on the SEIPS 2.0 system categories (e.g., tools/technology, tasks, environment, people/teams) [35]. All outcome data were analyzed to determine convergence of themes and repeated expression of reoccurring constructs. Discrepancies in the codes and labeling of themes were discussed by the core project team regularly and resolved; with themes simplified and altered accordingly until complete agreement was reached. Saturation of emergent themes was reached across > 400 simulation sessions provincially. The number of learners/participants and simulation sessions was collected using a standardized intake spreadsheet and was collated biweekly.

## Organizational learning

When using a simulation for systems integration approach, the collection and sharing of systems issues and learnings is key to informing a parent organization. The eSIM program’s infrastructure and approach was critical in a pandemic situation, as the information and learnings from the SIS were used to iteratively make improvements and help teams across the organization anticipate and plan for issue mitigation and process improvement. The timely analysis of large volumes of data that was being collated and themed in real time by the eSIM response team proactively informed and enabled scaling up of quality improvement activities across sites, departments, and zones. Our organizational learning included broad sharing of weekly dashboard updates of key outcomes, webinars (local and international webinars shared across the entire AHS organization and beyond resulting in over 4000 unique views; recording shared with > 100,000 email addresses) [4, 42], and key COVID-19 resources developed for simulation.

## COVID-19 simulation project key outcomes

### Highest impact and highest frequency outcomes

**Fig. 2:**
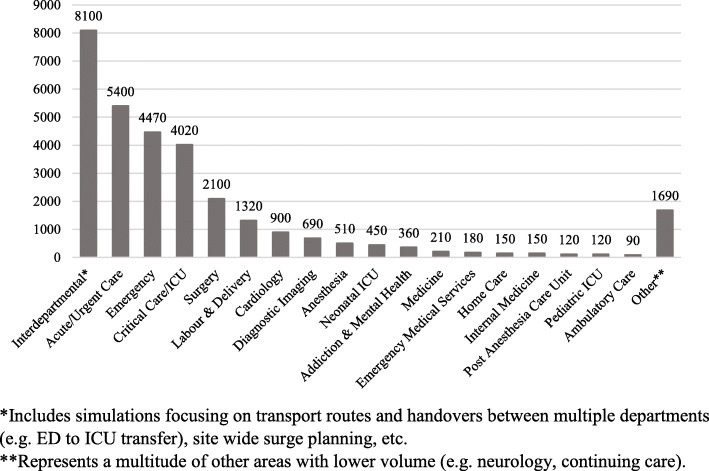
Estimated number of participants in COVID-19 preparedness simulation by department

The provincial eSIM COVID-19 simulation response team facilitated simulation session requests by leading the intake, design, development, planning, and facilitation of over 400 acute care simulation sessions across all sectors of our provincial health program. In under a 5-week period, an estimated 30,000 learners participated in simulation in both rural (17%) and urban (83%) centers across the province (Fig. 2).

There was a wide variance in the number of sessions/learners per request ranging from 1-3 days of simulation training and 10-400 people requiring training per request. Well over 2500 systems issues, including LSTs, have been proactively identified and mitigated through > 400 simulation sessions. Systems issues across all sessions were categorized into processes (61%), tools and technology (14%), persons and tasks (14%), environment (7%), and organization (4%).

Table 3 highlights the highest priority themes discussed in the debriefings (impact) and the highest reported outcomes (frequency) that emerged from all COVID-19 simulations.

**Table 3:** Highest impact and highest frequency outcomes

Key themes and qualitative outcomes (highest impact and highest frequency) identified in simulation	Systems categories
***1. Theme: Safe doffing (removal of PPE safely and in correct order)***	*People/teams/tasks;* *Tools/technology*
**Key outcomes**	
Cross monitor team members during doffing	
Use and IPAC poster as a cognitive aid	
Ensure “1 to 1” doffing to avoid breaches observed when too many doffing at once (e.g., getting ahead or behind in doffing sequence)	
Consistent role of a “PPE Coach” to support safe doffing-ensure focus and intention with every step	
Implement “just-in-time” review of safe doffing to reduce cognitive load during long stressful periods in PPE.	
***2. Theme: Conducting environmental scans of care areas is crucial in anticipating, planning ahead, and developing area processes***	*Environment;* *Tools/technology;* *People/Teams/Tasks*
**Key outcomes**	
Remove visitor chairs, extra equipment and linens from room to avoid waste, and additional cleaning between patients	
Keep transport routes	
Post signage for direction and decrease of clutter	
Creation of supply restocking checklist white this	
Creation of COVID-19 specific cart of required supplies	
Creation of small, labeled packages of specific supplies, or medications for fast grab and go	
Ensure team members are aware of the responsibilities required to maintain the space	
Ensure cleaning processes for removal of equipment leaving COVID-19 rooms (e.g., stretchers, wheelchairs)	
***3. Theme: Conduct inter-departmental/inter-hospital transport routes to establish communication and process between departments and professions***	*People/teams/tasks;* *Environment;* *Tools/technology*
**Key outcomes**	
Test and walk through the route	
Use signage if COVID-19 routes differ from the usual process	
Clean hallways of clutter and reduce traffic if possible	
Consider dedicating elevator banks for COVID-19 patients, staff and carts	
Establish a designated clean person on transports to ensure surfaces are cleaned (e.g., floors, elevator buttons, stretchers, and wheel chairs)	
Emergency medical services should use a common Stem in communication and pages: This line is supposed to be with the one below to read: "Emergency medical services should	
“Possible/Confirmed COVID-19 patient” this goes afte the word "pages" in line above	
Upon arrival of out of external hospital emergency medical services, ensure transport is ready and routes are prepared. white this Should read; Upon arrival of externa;l hospital emergency medical services, ensure transport is ready and routes are prepared.	
***4. Theme: Maintenance of isolation environment/prevention of contamination***	*Tools/technology;* *People/teams/tasks*
**Key outcomes**	
Removal of stethoscopes, phones, ID badges, lanyards, watches, and earrings from person prior to donning.	
When items are on person, reinforce learnings re: do not reach below gown for ID badge/pager/mobile phone; or under visor to adjust goggles/mask.	
Creation of bins on an external cart in donning area for dropping items into	
Keep numbers of staff in the room low when possible	
Ensure cleaning process for roving items such as clipboards, ultrasound machines, etc.	
***5. Theme: Roles and responsibilities***	*People/teams/tasks;* *Environment*
**Key outcomes**	
A runner role is needed across multi areas: Operating Room, Emergency Department, Labor and Delivery Unit, Intensive Care Unit (team member to bring supplies between isolated COVID-19 care area and non-isolated area)	
Consider the involvement of HCAs and Unit Clerks to bring necessary equipment required for teams	
Establish “clean” and “dirty” sides between rooms and within rooms by taping the floors for a visual cue	
Establish CODE COVID-19 team to attend to all rapid deteriorating patients	
***6. Theme: Innovative approaches to communication***	*Tools/technology;* *People/teams/tasks*
**Key outcomes**	
Use of dry erase markers on the shared glass wall of isolation to ante room	
Use of a laminated page that can be flipped back and forth	
Use of white boards to communicate key messages to outside team members	
Use of two-way radios (e.g., walkie talkies) and baby monitors	
Limit the use of negative pressure rooms and use ante rooms where available	
Use of speaker phone setting	
Use of tape on floor to communicate ‘clean versus dirty’ zones	
Check that monitors and speakers on phones (especially with PPE on) can be heard	
Include name/role tag stickers on outer PPE to ensure role clarity and effective communication	
Reduce noise and ensure use of closed-loop communication (additional communication challenges with PPE on)	
Use of trigger scripts on pagers to signal a priority response. Scripts like “COVID airway” or “COVID transport” to alert a team and get the right people and the right equipment to the right place.	
***7. Theme: Psychological safety and speaking up***	*People/teams/tasks*
**Key outcomes**	
Use critical language when breeches in PPE or when overcrowding in rooms occur	
Encourage all team members to speak up when they see breaches in safe PPE practices	
Removing hierarchical barriers can be challenging; promoting psychology safety is important for a cohesive team	
Go beyond your professional role to cross teach about PPE	
***8. Theme: Critical care medicine pre-intubation cognitive aid***	*People/teams/tasks;* *Tools/technology;* *Organization*
**Key outcomes**	
Communicate a plan ahead to ensure staff know their roles	
Double-check proper PPE during intubation	
Most experienced practitioner should perform the intubation	
Ensure the ventilator and video laryngoscopy device are in the room prior to start	
Consider back-up plan depending on available resources	
Ensure correct bagger filter is attached	
***9. Theme: Use of cognitive aids and checklists***	*Tools/technology*
**Key outcomes**	
Consider human factors science in the development of new COVID-19 cognitive aids and checklists	
Cognitive aids can be made into posters, use larger font, central point of reference white this	
They should be clear, easy to use adaptable to context, trained prior to implement, and pilot tested prior to use on a real patient	
Examples: COVID-19 airway pause checklist, checklists for buckets, and carts/bins, IPAC donning and doffing poster white this	

In analyzing the nine themes, it is clear that the rapid knowledge translation of best practices, new guidelines, and processes following system simulation events can potentially serve other organizations that may seek to learn from our centralized, coordinated approach to using system integration simulation for pandemic planning and preparedness.

In synthesizing the learnings from the highest impact and highest frequency outcomes of the 9 themes reported in Table 3, there are several reasons on why these salient themes came together based on our unique context and organizational level approach to SIS. Each of these themes informed our organization as to what was working well at one site, department, and zone and what needed to be improved and scaled up across the organization. For example, although we had an IPAC team, and multiple related resources and cognitive aids designed to support IPAC processes; it became clear, as identified by the frontline through simulation, that more needed to be done to address the nuances and unique clinical practices of IPAC specific to the pandemic. While hundreds of learners continued to struggle with safe doffing, simulation was able to inform the development of emerging best practices surrounding 1:1 doffing, what questions were repeatedly being discussed; why breaches were happening; and ensuring one person guided and supported individuals during doffing. Observing the trending of process-related systems issues enabled our team to share this finding broadly and focus our simulation tools and approaches to further address systems issues that impacted not just one unit, site or department enabling the learnings identified through simulation to be scaffolded and shared across the entire province. This led to further development of a systems-based process walk through approach, with debriefing, to ensure a systematic identification of process issues.

Repeated SIS debriefings where frontline team members asked about personal items such as watches, earrings, and identification badges in COVID rooms allowed us to share rapidly evolving strategies to address proper maintenance of isolation environments and the prevention of contamination. For teams who were struggling with effective ways to communicate outside of COVID rooms, we were able to rapidly share innovations on a large scale to any other teams who were in the same position in preparing their unit and staff to respond to the evolving nature of the global pandemic. This involved designing, through simulation, innovative approaches necessary to keep staff and patients safe by communicating using dry erase markers on glass between rooms, as one example. As protected COVID-19 intubations were identified as a high anxiety and high-risk time for healthcare workers, as shown by repeated simulation requests from healthcare staff targeting these objectives, having the ability to test these processes in simulation and refine a critical care medicine cognitive aid for pre-intubation and then share these findings on such a large scale was essential to successful preparation and safety of staff. As a result of multiple simulations on intubation, a COVID-19 cognitive aid was developed, which was then contextualized for rural sites and then broadly shared across the province using our coordinated system.

## Discussion

Our use of simulation for systems integration involved taking a project approach to the initiation, planning, execution, and reporting [39] (e.g., shared learnings) to enable proactive improvement work for safer, more efficient, and reliable care processes for patients and healthcare teams [43–50]. This paper adds to the emerging literature on using simulation for pandemic planning and preparedness [8, 9] as it describes a highly coordinated COVID-19 pandemic simulation response using a centralized team, robust valid curriculum, and data outcomes team to analyze and rapidly share an unprecedented volume of simulation data for a large-scale SIS project across the largest single health authority in Canada.

Unfortunately, the speed at which the COVID-19 pandemic has emerged in Canada may prevent every acute care center in both urban and rural settings in having healthcare teams participate in “just-in-time” in situ simulation-based learning and systems/process. The ability to test new pandemic processes, and share organizational learnings to be disseminated on a mass scale has been pivotal in preparing our healthcare system to respond to the emergent threat of COVID-19.

The importance of using simulation as a “first choice” strategy for ensuring individual, team, and system readiness in times of crisis is supported by multiple publications in the literature [5, 51–53], and highlights that a sustained investment in simulation programs will have immeasurable impacts across healthcare systems following the pandemic. Many of these papers [3, 6–9] indicate a single site or unit approach to pandemic preparation, although do not specifically highlight using a coordinated and centralized simulation team, development of robust valid curriculum, and real time data analysis of emerging themes from hundreds of simulations. Although “more” may not necessarily always be better, we advocate that during times of pandemic where time sensitivity and reliable information are of utmost importance, a coordinated organizational-wide simulation response approach allows for broader and faster sharing, scalability, and generalizability of the findings. The validation of these 9 themes from simulations across multiple sites and teams outweighs the findings at only one.

One of the emerging findings that differed our project from other COVID-19 system simulations experiences both nationally and globally in using simulation as both a learning and evaluation tool to prepare [3, 6–9], was recognizing the critical importance of embedding simulation as a central part of the organizational learning, and the overall pandemic preparedness strategy. Similar to an operational “Emergency Command Center,” simulation programs (regardless of the size) need to situate themselves to be members “at the table” with key programs informing decision-making, and influencing organizational planning and pandemic preparedness from the beginning. Many factors influence the ability of simulation programs to “take this seat” including how the program is established prior to pandemic and how well it may be recognized by hospital administration staff in informing the organization of the needs and challenges coming directly from the frontline care providers. We situated ourselves as key informants to leaders and the organization’s administration, through planning and executing a large-scale simulation for systems integration project. Our outcomes, which identified over 2500 systems issues, including LSTs, had meaning to the organization because they were widespread and converged data from several sites including both urban and rural centers and across the continuum of care. In addition, we could equally share and reach all sites within our organizational structure for the new emerging themes we were uncovering in real time.

We informed our organization on multiple levels through our webinars, dashboards, and relationships built across a large health authority. Queueing IPAC team members of ongoing concerns with best practices for safe doffing to enable targeted approaches; sharing weekly questions still arising from the frontline to the IPAC team; and then supporting the development of a “doffing buddy” program are a few examples. This example highlights that while the emerging literature on COVID-19 simulations [3, 6-9] differ in their approach and scale of SIS, there is overlapping outcomes and triangulation of findings specific to IPAC across the different systems, countries, and jurisdictions. Essentially, this underlines the validity and robustness of the use of simulation for systems integration methodology for organizational learning.

We realized that process-related systems issues were widespread and the requests for environmental scans with debriefing prompted hundreds of teams to start re-designing their COVID care spaces after one of our “shared learnings” webinars. Seeing the rapid uptake of teams developing checklists and cognitive aids in isolation of each other, and with limited human factors experience, led us to establish collaborative human factors tools on how to develop an effective cognitive aid and principles of safe use. We believe all of these developments were the result of a swift and purposeful large-scale centralized approach.

Recognizing that not all health authorities have opportunity to coordinate or operationally support a centralized team and curriculum across sites; we recommend the explicit effort of simulation programs to align with other programs in meaningful ways to analyze and share emerging data in real-time to support validation for broader sharing and scalability when possible.

The simulation methods and outcomes used in the COVID-19 response have had a profound impact on our teams, processes, and system functioning. Our experience offers other organizations’ learnings to glean and consider the importance of establishing a centralized simulation response team for scale, spread and speed of knowledge translation, implementation, and change management effectiveness. With on-going use of SIS sessions informing cycles of improvement, the simulation will continue to be a key organizational tool we will use to further manage this evolving crisis, as well as future needs of our healthcare organization. Our pandemic preparedness highlighted the essential use of simulation as both an evaluation tool capable of testing systems and processes, and identifying and mitigating LSTs, as well as an education tool capable of rapidly preparing frontline teams in terms of the changes identified above. This project has identified how the dissemination and broadcasting of curriculum and lessons learned (e.g., emerging themes, innovations, systems-based approaches) from simulation can rapidly help a large organization over a large geographic area be adequately prepared for an evolving situation like the COVID-19 pandemic of 2020.

## Supplementary information

**Additional file 1:.** eSIM COVID simulation requests: Questions to guide needs of end user

**Additional file 2:.** Walkthrough process simulation

## Data Availability

Data sharing is not applicable to this article as no datasets were generated or analyzed.

## Abbreviations

AHS: Alberta Health Services
COVID: Coronavirus disease
COVID-19: Coronavirus disease 2019
eSIM: educate Simulate Innovate Motivate
HCA: Health care aides
HCP: Healthcare provider
HF: Human factors
ID: Identification badge
IPAC: Infection prevention and control
LST: Latent safety threat
PPE: Personal protective equipment
SEIPS: System engineering initiative for patient safety
SFD: System focused debriefing
SIS: Simulation for systems integration
