# Correction to: COVID-19 pandemic preparation: using simulation for systems-based learning to prepare the largest healthcare workforce and system in Canada

**DOI:** 10.1186/s41077-021-00165-1

**Published:** 2021-04-20

**Authors:** Mirette Dubé, Alyshah Kaba, Theresa Cronin, Sue Barnes, Tara Fuselli, Vincent Grant

**Affiliations:** 1 eSIM Provincial Simulation Program, Alberta Health Services, Alberta Health Services, 1403 29th Street NW, Calgary, Alberta T2N 2 T9 Canada; 2 Department of Community Health Sciences, Cumming School of Medicine, University of Calgary, Calgary, Canada; 3 Departments of Pediatrics and Emergency Medicine, Cumming School of Medicine, University of Calgary, Calgary, Canada; 4 KidSIM Pediatric Simulation Program, Alberta Children’s Hospital, 28 Oki Dr NW, Calgary, Alberta T3B 6A8 Canada

**Correction to: Adv Simul 5, 22 (2020)**

**https://doi.org/10.1186/s41077-020-00138-w**

The original article [1] contains copyediting errors in Table 3. The correct presentation of Table 3 can be viewed ahead in this Correction article.

**Table 3:** Key themes and qualitative outcomes (highest impact and highest frequency) identified across simulations

Key Themes and Qualitative Outcomes (Highest Impact and Highest Frequency) identified in Simulation	Systems Categories
***1. Theme: Safe doffing (removal of PPE safely and in correct order)***	*People/Teams/Tasks; Tools/Technology;*
**Key Outcomes:**	
• Cross monitor team members during doffing	
• Use an IPAC poster as a cognitive aid	
• Ensure “1 to 1” doffing to avoid breaches observed when too many doffing at once (e.g. getting ahead or behind in doffing sequence)	
• Consistent role of a “PPE Coach” to support safe doffing- ensure focus and intention with every step	
• Implement “just-in-time” review of safe doffing to reduce cognitive load during long stressful periods in PPE.	
***2. Theme: Conducting environmental scans of care areas is crucial in anticipating, planning ahead and developing area processes***	*Environment* *Tools/Technology;* *People/Teams/Tasks*
**Key Outcomes:**	
• Remove visitor chairs, extra equipment and linens from room to avoid waste and additional cleaning between patients	
• Keep transport routes clear	
• Post signage for direction and decrease of clutter	
• Creation of supply restocking checklist	
• Creation of COVID-19 specific cart of required supplies	
• Creation of small, labelled packages of specific supplies or medications for fast grab and go	
• Ensure team members are aware of the responsibilities required to maintain the space	
• Ensure cleaning processes for removal of equipment leaving COVID-19 rooms (e.g stretchers, wheelchairs)	
***3. Theme: Conduct Inter-Departmental/Inter-Hospital Transport routes to establish communication and process between departments and professions***	*People/Teams/Tasks;* *Environment;* *Tools/Technology*
**Key Outcomes:**	
• Test and walk through the route	
• Use signage if COVID-19 routes differ from usual process	
• Clean hallways of clutter and reduce traffic if possible	
• Consider dedicating elevator banks for COVID-19 patients, staff and carts	
• Establish a designated clean person on transports to ensure surfaces are cleaned (e.g. floors, elevator buttons, stretchers, wheel chairs, etc.)	
• Emergency Medical Services should use a common pager Stem: “Possible/Confirmed COVID-19 patient”	
• Upon arrival of out of hospital emergency medical services, ensure transport is ready and routes are prepared.	
***4. Theme: Maintenance of Isolation Environment/Prevention of Contamination***	*Tools/Technology;* *People/Teams/Tasks*
**Key Outcomes:**	
• Removal of stethoscopes, phones, ID badges, lanyards, watches, and earrings from person prior to donning.	
• When items are on person, reinforce learnings re: don’t reach below gown for ID badge/pager/mobile phone; or under visor to adjust goggles/mask.	
• Creation of bins on an external cart in donning area for dropping items into	
• Keep numbers of staff in the room low when possible	
• Ensure cleaning process for roving items such as clipboards, ultrasound machines, etc.	
***5. Theme: Roles and Responsibilities***	*People/Teams/Tasks* *Environment*
**Key Outcomes:**	
• A runner role is needed across multi areas Operating Room, Emergency Department, Labour & Delivery Unit, Intensive Care Unit (team member to bring supplies between isolated COVID-19 care area and non-isolated area)	
• Consider the involvement of HCAs and Unit Clerks to bring necessary equipment required for teams	
• Establish “clean” and “dirty” sides between rooms and within rooms by taping the floors for a visual cue	
• Establish CODE COVID-19 team to attend to all rapid deteriorating patients	
***6. Theme: Innovative Approaches to Communication***	*Tools/Technology* *People/Teams/Tasks*
**Key Outcomes:**	
• Use of dry erase markers on the shared glass walls between isolation to ante room	
• Use of a laminated page that can be flipped back and forth	
• Use of white boards to communicate key messages to outside team members	
• Use of two-way radios (e.g. walkie talkies) or baby monitors	
• Use of speaker phone setting	
• Use of tape on floor to communicate ‘clean versus dirty’ zones	
• Check that monitors and speakers on phones (especially with PPE on) can be heard	
• Include name/role tag stickers on outer PPE to ensure role clarity and effective communication	
• Reduce noise and ensure use of closed loop communication (additional communication challenges with PPE on)	
• Use of trigger scripts on pagers to signal a priority response. Scripts like “COVID airway” or “COVID transport” to alert a team and get the right people and the right equipment to the right place.	
***7. Theme: Psychological Safety and Speaking Up***	*People/Teams/Tasks*
**Key Outcomes:**	
• Use critical language when breeches in PPE or when overcrowding in rooms occur	
• Encourage all team members to speak up when they see breaches in safe PPE practices	
• Removing hierarchical barriers can be challenging; promoting psychology safety is important for a cohesive team	
• Go beyond your own professional role to cross teach about PPE	
***8. Theme: Critical Care Medicine Pre-Intubation Cognitive Aid***	*People/Teams/Tasks;* *Tools/Technology;* *Organization*
**Key Outcomes:**	
• Communicate a plan to ensure staff know their roles during intubation	
• Double-check proper PPE during intubation	
• Most experienced practitioner should perform the intubation	
• Ensure the ventilator and video laryngoscopy device are in the room prior to start	
• Consider back-up plan depending on available resources	
• Ensure correct bagger filter is attached	
***9. Theme: Use of Cognitive Aids and Checklists***	*Tools/Technology*
**Key** ***Outcomes***	
• Consider human factors science in the development of new COVID-19 cognitive aids and checklists	
• Cognitive aids can be made into posters, use larger font, central point of reference	
• They should be clear, easy to use, adaptable to context, staff trained on prior to implementation and pilot tested prior to use on a real patient	
• Examples: COVID-19 Airway pause checklist, checklists for buckets and carts/bins, IPAC Donning & Doffing Poster	

## References

[1] Dubé M (2020). COVID-19 pandemic preparation: using simulation for systems-based learning to prepare the largest healthcare workforce and system in Canada. Adv Simul.

