# Supplementary material for: COVID-19 pandemic preparation: using simulation for systems-based learning to prepare the largest healthcare workforce and system in Canada
Source: Adv Simul (Lond). 2020 Aug 18;5:22. doi: 10.1186/s41077-020-00138-w (PMC7432586; doi:10.1186/s41077-020-00138-w)
Supplement: Supplementary file 1 — Additional file 1:. eSIM COVID simulation requests: Questions to guide needs of end user [file 41077_2020_138_MOESM1_ESM.docx]

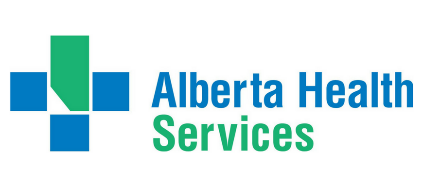

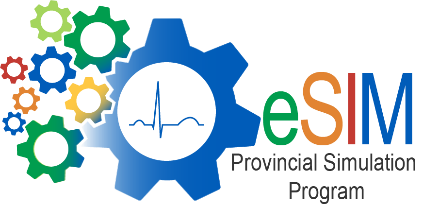


**eSIM COVID Simulation Requests: Questions to Guide Needs of End User**

1. Requestor

Click here to enter text.

1. Requestor email and contact information

Click here to enter text.

1. Site/Department

Click here to enter text.

1. Acute/Rural/Primary

Click here to enter text.

1. Date Sim Required

Click here to enter a date.

1. Objectives for COVID Simulation (may be several)

Recognition Medical management

Process (i.e. transport, triage, table top exercise) Procedure (ie intubation)

Donning/Doffing Teams Roles/Responsibilities

Other: Click here to enter text.

1. Staff for Training:

Frontline Education

Educator Management leadership

1. Pre Learning Education Completed

IPC D and D

COVID area specific

1. Number of Staff/Physician to be trained

Click here to enter text.

1. Time Allocated

Click here to enter text.

1. Insitu Classroom
2. Supplies/Equipment available

Click here to enter text.

1. Internal Stakeholders Needed

IPC WHS

MOH EDM

Protection Services Environmental Services

Other: Click here to enter text.

1. Reiterate eSIM role vs End User Role
2. Scenario development needed: Yes No
3. Resources shared with end users: Yes No
4. Metrics & Session info sent to CoVID Team: Yes No
