# Supplementary material for: COVID-19 pandemic preparation: using simulation for systems-based learning to prepare the largest healthcare workforce and system in Canada
Source: Adv Simul (Lond). 2020 Aug 18;5:22. doi: 10.1186/s41077-020-00138-w (PMC7432586; doi:10.1186/s41077-020-00138-w)
Supplement: Supplementary file 2 — Additional file 2:. Walkthrough process simulation [file 41077_2020_138_MOESM2_ESM.pdf]

Walkthrough Process Simulation

What is it? A physical walkthrough-style simulation of a scenario/case in the live environment with key participants. Purpose is to find latent safety threats and determine how the process with a Covid-19 patient differs from your area's day to day process. A walk through supports team and individual staff confidence and readiness to provide safe patient care during the pandemic.

Steps of a Walkthrough:

1) **Include appropriate experts and stakeholders.** Managers, educators, nurses, physicians, RRTs, environmental services, Protective services, IP&C. Bring cognitive aids/checklists applicable to your area

2) **Determine objectives. Common objectives for COVID-19 Simulation:**
   - Establish new COVID-19 processes and workflows from triage to admission
   - Apply new COVID-19 processes and medical management to a deteriorating patient
   - Establish new roles and process involved with inter-hospital transport of a COVID-19 patient

3) **Prebriefing:** Review objectives, goal is to find latent safety threats and assess process. Focus on process, not the medicine. May be "stop and go" as you work through transitions in care, with timeouts for discussion and clarification. Each observer will speak to their role and the action occurring at each step in the process.

4) **Physical Walkthrough** of your area. Complete environmental scan for barriers, missing equipment etc. Ask for feedback from all team members. Reflect on day-to-day practice/process. Organize these findings. (See System Integration Categories below).

5) **Debriefing:** Discuss and share findings, assign action items to be addressed. How will you implement changes? How will you share information with your team(s)? Take a systems approach to debriefing the highest risk, highest frequency items. Consider tools, technology, environment, tasks, processes, people and organization (See Table 1).[1]

Table 1: Walkthrough Simulation Process: System Categories[2]

**TOOLS and TECHNOLOGY**	**ENVIRONMENT**
• Airway carts, Covid Carts, Supply buckets, pre-packages<br>• 2 way radios, phones, baby monitors, Vocera<br>• Equipment, PPE checklists, posters and cognitive aids<br>• Computers, IT, patient charts<br>• Paging systems	• Physical set up and layout<br>• Barriers<br>• Signage<br>• Transport Routes and wayfinding
**TASKS**<br>• Donning and Doffing<br>• PPE Coach<br>• Complexity of patient care and assessments	**PROCESSES**<br>• Communication pathways<br>• Workflow<br>• Policies (testing of new or existing)
**PEOPLE**<br>• Roles and responsibilities<br>• Team process changes<br>• New response teams	**ORGANIZATION**<br>• Coordination of teams<br>• Training<br>• Policies<br>• Resource availability

***Disclaimer: these resources may be contextual for specific needs/groups. As the pandemic rapidly evolves, these resources, information and tools shared **may also become obsolete**. Please ensure checking for the most relevant and recent resources.*

1. Dubé MM, Reid J, Kaba A, Cheng A, Eppich W, Grant V, et al. PEARLS for Systems Integration: A Modified PEARLS Framework for Debriefing Systems-Focused Simulations. Simul Healthc J Soc Simul Healthc [Internet]. 2019 May [cited 2019 Jun 22]; Available from: http://europepmc.org/abstract/med/31135684
2. Holden RJ, Carayon P, Gurses AP, Hoonakker P, Hundt AS, Ozok AA, et al. SEIPS 2.0: a human factors framework for studying and improving the work of healthcare professionals and patients. Ergonomics. 2013 Nov 1;56(11):1669–86
